# Establishment and validation of a callus tissue transformation system for German chamomile (*Matricaria chamomilla* L.)

**DOI:** 10.1186/s12870-023-04680-3

**Published:** 2023-12-20

**Authors:** Yuling Tai, Jie Zhang, Youhui Chen, Yi Yuan, Honggang Wang, Luyao Yu, Shuangshuang Li, Lu Yang, Yifan Jin

**Affiliations:** School of Life Science, Anhui Agricultural University, Hefei, 230036 People’s Republic of China

**Keywords:** German chamomile, Callus tissue transformation system, *McFPS*, Gene function

## Abstract

**Background:**

German chamomile (*Matricaria chamomilla* L.) is an important medicinal plant, and the essential oils in the flowers have various biological activities. Genetic transformation systems are important for plant quality improvement and molecular research. To the best of our knowledge, a genetic transformation system has not yet been reported for German chamomile.

**Results:**

In this study, we developed *Agrobacterium*-mediated transformation protocols for German chamomile callus tissues. This involved optimizing key parameters, such as hygromycin and cefotaxime concentrations, bacterial density, and infection and co-culture durations. We also performed gas chromatography–mass spectrometry analysis to identify volatile compounds in non-transgenic and transgenic callus and hairy root tissues. Furthermore, to compare and verify the callus transformation system of German chamomile, we transferred *McFPS* to the hairy roots of German chamomile. The results showed that the optimal conditions for *Agrobacterium*-mediated callus tissue transformation were as follows: explant, petiole; cefotaxime concentration, 300 mg/L; hygromycin concentration, 10 mg/L; and bacterial solution concentration, OD_600_ = 0.6; callus transformation efficiency was the highest when the co-culture time was 3 days.

**Conclusions:**

Establishment of a high-efficiency callus transformation system will lay the foundation for gene function identification in German chamomile.

**Supplementary Information:**

The online version contains supplementary material available at 10.1186/s12870-023-04680-3.

## Background

German chamomile (*Matricaria chamomilla* L.) is an annual aromatic medicinal plant with anti-inflammatory, analgesic, anti-allergic, and antioxidant properties [1, 2]. German chamomile is native to Europe, and it is mainly cultivated in certain regions of China [3]. The active ingredients of German chamomile are sesquiterpenoids from essential oils, for example, bisabolol, which has anticancer activity; (*E*)-β-farnesene (EβF), which is involved in defense chemical signaling of aphids; and nerolidol, which is commonly used in fragrance and flavor industries. In addition, the main active substances of German chamomile essential oils usually include γ-muurolene, β-santalol, δ-selinene, *cis*-β-farnesene, and α-guaiene. Sesquiterpenoids belong to the terpene family of molecules, and they are formed by the polymerization of three molecules of isoprene with a skeleton containing 15 carbon atoms [4–7]. The biosynthetic pathway of sesquiterpene compounds is complex [8]. In plants, the synthetic pathways of sesquiterpenoids are the mevalonate (MVA) and 2-*C*-methyl-d-erythritol 4-phosphate pathways (MEP) [9, 10].

Farnesyl pyrophosphate synthase (FPS) is a type of allyltransferase that synthesizes farnesyl diphosphate (FPP) from isoprenoyl pyrophosphate (IPP) and geranyl pyrophosphate (GPP) [11, 12]. FPP is the C15 backbone of many sesquiterpenoids, so FPS is an important rate-limiting enzyme for sesquiterpenoid metabolism [13, 14]. To date, *FPS* has been successfully cloned and studied in various plants. For instance, Chen performed knockdown of *FPS* genes by using the RNAi technique, and the results showed that the *FPS* genes are involved in the production of EβF [15]. Closa et al. performed knockdown experiments and found that, when *FPS* is not expressed, embryos appear stalled during early development in *Arabidopsis* and cause damage to gene inheritance; this suggests that *FPS* has a critically important effect on *Arabidopsis* growth and development [16].

The *Agrobacterium*-mediated genetic transformation system can infiltrate plant wounds under natural conditions and stably integrate exogenous DNA (T-DNA) from Ti plasmids into the genome of recipient plants [17, 18]. Currently, the *Agrobacterium*-mediated method is used in a variety of plants because of its simplicity, rapidity, stability, and effectiveness [19, 20]. However, changes in each factor, such as antibiotic concentration, bacterial solution concentration, infestation time, co-culture time, and exogenous genes, can have an impact on the efficiency of genetic transformation. Stevens and Pijut established the genetic transformation of broadleaf tree pumpkin ash by using the *Agrobacterium*-mediated method and found that the growth of *Agrobacterium* can be significantly inhibited at a concentration of 400 mg/L timentin and 20 mg/L kanamycin [21]. Yang et al. studied the conditions for obtaining transgenic *Phellodendron* bark and found that the highest transformation efficiency was achieved when the preculture time was 2 days [22]. Belarmino and Mii established the *Phalaenopsis* transformation system with two strains of *Agrobacterium*, LBA4404 and EHA101, and the transformation efficiency increased significantly when the explants were co-cultured with the strains for 10 h [23]. Karmakar analyzed the conditions of pigeon pea transformation system and found that the chances of obtaining resistant seedlings were the highest when *Agrobacterium* solution had OD_600_ = 0.25 and the infestation time was 15 min [24]. *Asteraceae* is a family of common plants with various species and different functions. Generally, the family can be divided into two categories: ornamental plants, such as golden hydrangea and dahlia, and medicinal plants, such as *Boletus*, German chamomile, *Artemisia*, and snowdrop. To acquire species of excellent quality, researchers have started to conduct research on the genetic transformation of Asteraceae. For example, Gupta and Ur Rahman established an efficient transgenic system with hypocotyl explants of marigold and screened it using polymerase chain reaction (PCR) and protein blotting techniques [25].

To the best of our knowledge, a callus transformation system for German chamomile has not yet been reported, and the use of callus transformation for the functional validation of German chamomile genes could be an effective approach. In the *Agrobacterium*-mediated callus tissue transformation system, the type of explant, hormone ratio, and *Agrobacterium* species all affect the callus tissue induction rate, and the positive strain acquisition rate can be improved by optimizing the induction and transformation conditions. Therefore, in this study, we used the petioles of German chamomile as explants, optimized the German chamomile callus induction system, transferred *McFPS* for validation, and established a stable callus genetic transformation system.

## Results

### Identification and cloning of *McFPS*

**Fig. 1:**
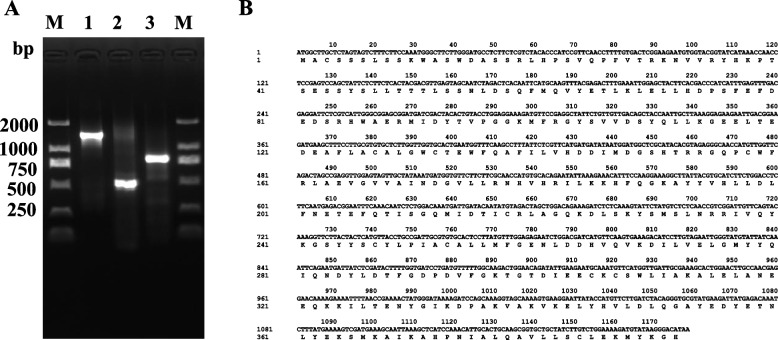
Agarose gel electrophoresis of *McFPS* clones (**A**) and *McFPS* sequence and encoding protein (**B**) Note: M: Standard molecular weight of DNA; 1: full length of *McFPS,* 2: 5′-RACE clone of *McFPS*; 3: 3′-RACE clone of *McFPS*

We identified a gene related to *FPS* from the German chamomile transcriptome database by using NCBI (https://www.ncbi.nlm. nih.gov/) BLAST and named it *McFPS*. Specific primers were designed on the basis of sequences from the German chamomile transcriptome database*.* After rapid amplification of cDNA ends (RACE) experiments, the coding sequences of *McFPS* were obtained (Fig. 1A). The full-length complementary sequence of *McFPS* is 1179 base pairs in length, encodes 392 amino acids with a molecular mass of 44.93 kDa (Fig. 1B).

### Optimization of hygromycin concentration for transgenic callus tissues of German chamomile

**Fig. 2:**
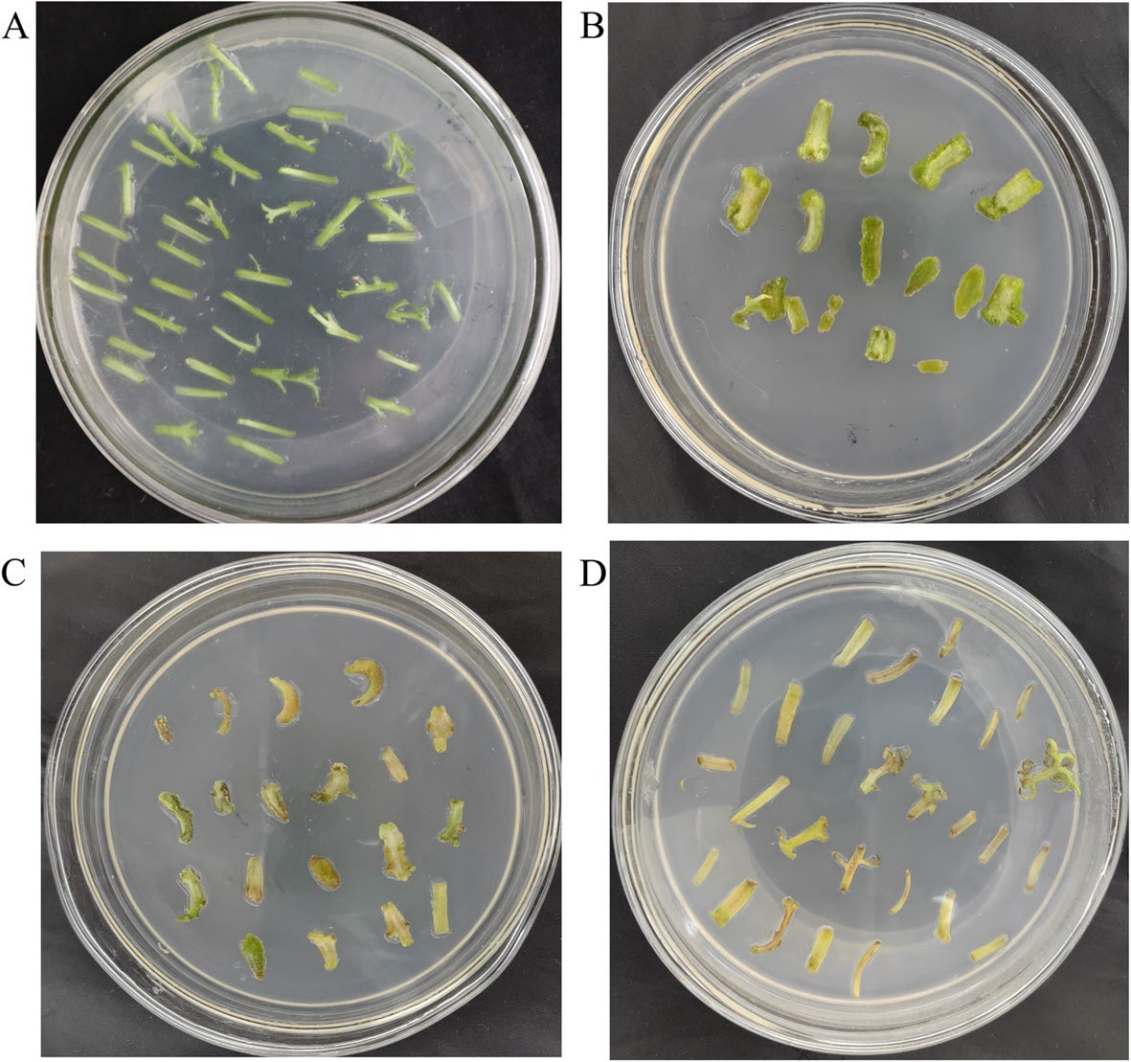
Effects of hygromycin on explant growth **A** Explants grown for 3 days (without Hyg). **B** Explants grown for 14 days (without Hyg). **C** Explants grown for 14 days (including 10 mg/L Hyg). **D** Explants grown for 14 days (including 15 mg/L Hyg)

Our preliminary results showed that the optimal callus induction medium (CIM) for German chamomile was Murashige and Skoog (MS) + 2.0 mg/L 6-benzylaminopurine (6-BA) + 0.4 mg/L naphthaleneacetic acid (NAA). To find a suitable concentration of hygromycin for callus tissue selection, German chamomile petioles were cultured on CIM containing different concentrations of hygromycin, and petiole callus regeneration and differentiation were observed. The callus tissue growth was severely inhibited when the concentration of hygromycin was increased. Within 14 days, German chamomile petioles on CIM without hygromycin continued to grow (Fig. 2A, 2B). However, when CIM contained 5 mg/L hygromycin, a slight decrease in growth was observed during the early stage of the culture and growth increased over time; this indicated that German chamomile was sensitive to hygromycin. At a hygromycin concentration of 10 mg/L, petioles began to exhibit browning and necrosis, leading to a browning rate of 69.66% (Fig. 2C). When the concentration of hygromycin was higher than 20 mg/L, petiole growth was inhibited, the degree of browning was serious (up to 91.22%), and finally the petiole died (Fig. 2D). Therefore, 10 mg/L hygromycin was a suitable screening concentration for the genetic transformation of German chamomile (Table 1).

**Table 1:** Effects of hygromycin concentration on transformation after 14 days of culture

Column number	Concentration of hygromycin (mg/L)	Inoculation number	Browning rate (%)
1	0	100	0
2	5	100	31.00 ± 4
3	10	100	69.66 ± 6.11
4	20	100	91.22 ± 1.33
5	30	100	100.00
6	40	100	100.00

### Effects of different concentrations of cefotaxime on *Agrobacterium tumefaciens* inhibition

Antibiotic sensitivity experiment is one of the key steps in the establishment of a genetic transformation system, and its main function is to screen plants and inhibit the growth of *A. tumefaciens*. To find a suitable concentration of cefotaxime to inhibit *A. tumefaciens*, German chamomile petioles were cultured on CIM containing different concentrations of cefotaxime. *A. tumefaciens* was severely inhibited when the concentration of cefotaxime was increased from 100 mg/L to 400 mg/L. When the concentration of cefotaxime was 300 mg/L, *A. tumefaciens* was completely inhibited (contamination rate, 4.67%), and the callus growth was good. When the concentration of cefotaxime was higher than 400 mg/L, *A. tumefaciens* was completely inhibited; however, petiole growth was inhibited and the petiole turned brown and finally the petiole died. Therefore, 300 mg/L cefotaxime was a suitable screening concentration for German chamomile genetic transformation, and it could completely inhibit the growth of *A. tumefaciens* without serious toxicity to the receptor materials (Table 2).

**Table 2:** Effects of cefotaxime concentration on transformation after 7 days of culture

Column number	Concentration of cefotaxime (mg/L)	Inoculation number	Contamination rate/Error (%)
1	100	100	96.33 ± 4.14
2	200	100	37.33 ± 7.50
3	300	100	4.67 ± 1.52
4	400	100	0
5	500	100	0

### Analysis of the infection solution concentration and infection duration

On the basis of the optimized concentrations of hygromycin and cefotaxime, different concentrations of *A. tumefaciens* (OD_600_ = 0.4, 0.6, and 0.8) were used to infect the petiole segments. After identification and analysis, the results showed that 32% transformation efficiency was achieved when OD_600_ was 0.4 (Table 3). Transformation efficiency was the highest (68.21%) when OD_600_ was 0.6. Transformation efficiency was 40.66% when OD_600_ was 0.8. Therefore, OD_600_ = 0.6 was the optimal concentration for transformation. In addition, infection duration is an important factor that affects the efficiency of genetic transformation. The transformation efficiency varied greatly when different infection durations were used. The highest transformation efficiency (68.21%) was achieved when petiole segments were infected for 5 min. The transformation efficiency was 48.72% when petiole segments were infected for more than 15 min, which was significantly lower than that obtained in 5 min. Therefore, 5 min may be the ideal duration for *A. tumefaciens* infection.

**Table 3:** Effects of infection solution concentration and infection duration on transformation

Column number	Concentration of bacterial solution (OD_600_)	Infection time (min)	Inoculation number	Transformation efficiency (%)
1	0.4	5	100	32.00 ± 2.64
2	0.6	5	100	68.21 ± 4.44
3	0.8	5	100	40.66 ± 2.08
4	0.6	10	100	67.18 ± 3.87
5	0.6	15	100	48.72 ± 4.7
6	0.6	20	100	35.90 ± 3.87

### Influence of co-culture duration on the culture of transgenic callus tissues of German chamomile

Co-culture is an important period in which T-DNA can be transferred into plant cells, and different co-culture durations have a certain impact on the transformation efficiency. Thus, to study the effects of different co-culture durations on the culture of transgenic callus tissues of German chamomile, the callus was cultured for 1, 2, 3, and 4 days by using the optimal hygromycin concentration, optimal cefotaxime concentration, infection solution concentration, and infection duration. The results showed that a few resistant callus tissues were obtained when co-cultured for 1 day and transformation efficiency was 12.33%, which indicated an insufficient period for the interactions between petiole segments and bacteria (Table 4). The expression rate of transgenic callus was the highest (65%) when co-cultured for 3 days. However, when the co-culture time was 5 days, overgrowth of *A. tumefaciens* resulted in browning at the end of some petiole segments, and the transformation frequency decreased (47.67%). Thus, 3 days of co-cultivation treatment was the best period for obtaining transgenic callus tissues of German chamomile.

**Table 4:** Effects of co-culture time on the culture of transgenic callus tissues of German chamomile

Column number	Co-culture days (day)	Inoculation number	Transformation efficiency（%）
1	1	100	12.33±2.52
2	2	100	16.33±4.04
3	3	100	65.00±7.55
4	4	100	47.67±2.52

### Morphological observation of resistant callus tissues

**Fig. 3:**
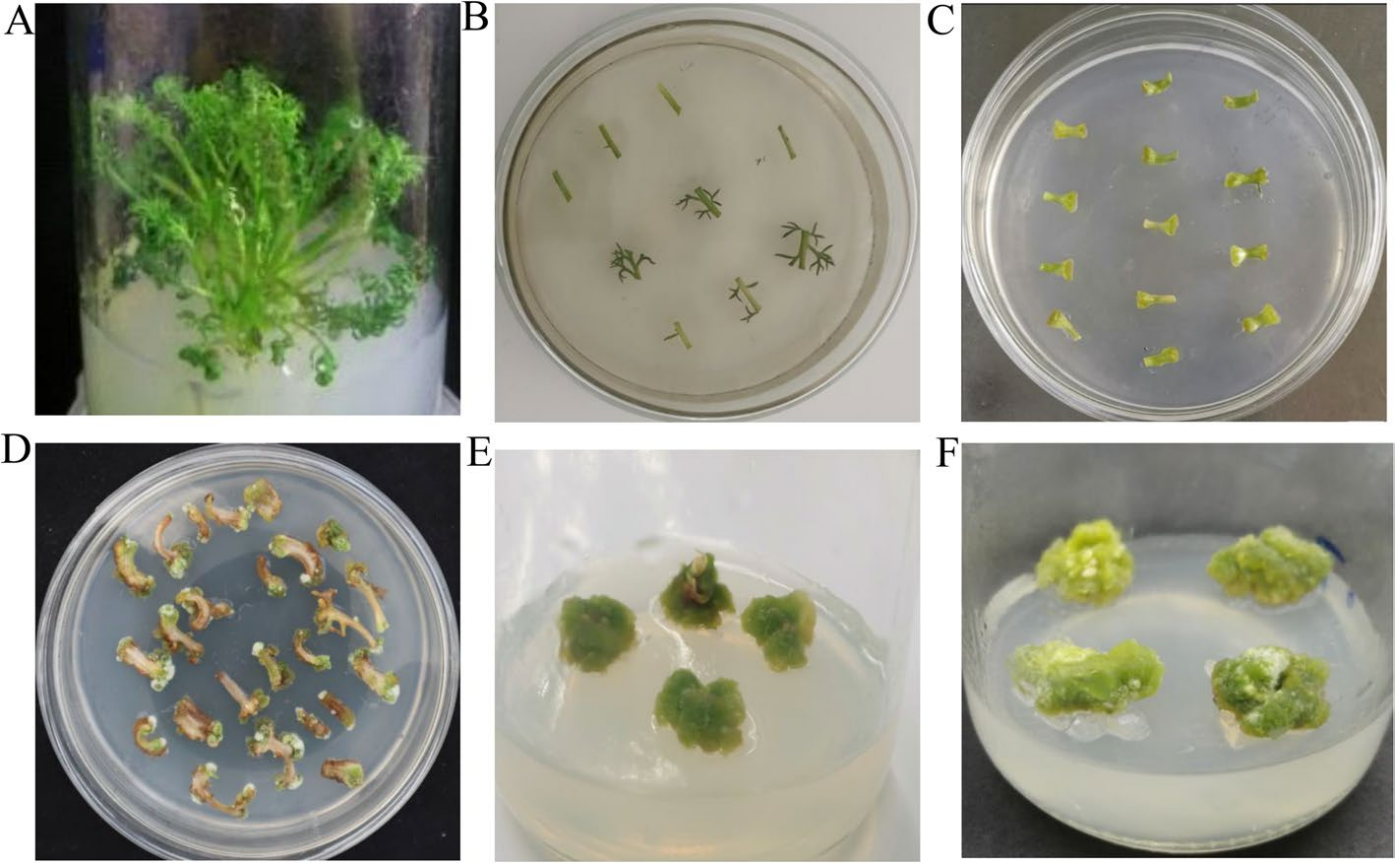
Growth of resistant German chamomile plants. **A** Sterile seedlings. **B** Explants of German chamomile grown for 3 days. **C** Explants of German chamomile grown for 10 days. **D** Explants of German chamomile grown for 20 days. **E** Screening of resistant callus tissues and explants of German chamomile grown for 30 days. **F** Screening of resistant callus tissues and explants of German chamomile grown for 40 days

According to the results, the screening culture medium for German chamomile resistant callus induction was MS + 2.0 mg/L 6-BA + 0.4 mg/L NAA + 300 mg/L cefotaxime + 10 mg/L hygromycin (Fig. 3A). When the petiole segments were cultured for 3 days, their morphology did not change significantly (Fig. 3B). After 10 days of culture, the petiole segments showed swelling and a small amount of callus was produced (Fig. 3C). When the explants were cultured for 20 days, some explants gave rise to a green callus-like mass. The parts that failed to transform showed browning and died (Fig. 3D). When the explants were cultured for 30–40 days, the resistant callus continued to grow (Fig. 3E, F).

### Identification and gas chromatography-mass spectrometry (GC–MS) detection of resistant callus tissues

**Fig. 4:**
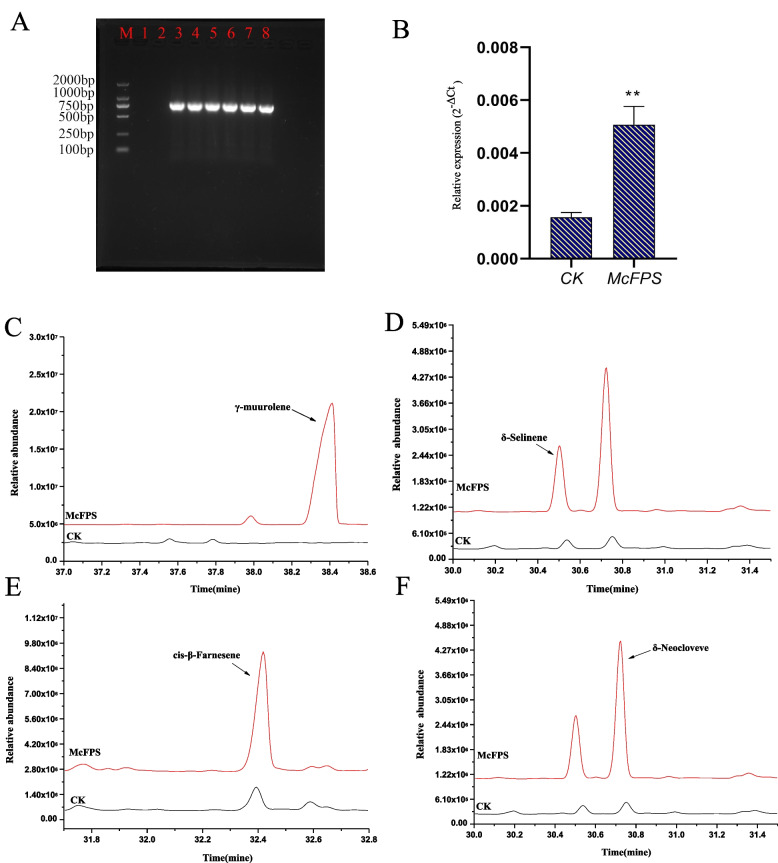
GC–MS analysis of transgenic callus tissues of *McFPS* **A** Verification of German chamomile resistant callus tissues by using hygromycin resistance gene primers. **B** qPCR analysis of transgenic callus tissues of *McFPS*. **C**–**F** Analysis of volatile components in transgenic callus tissues of *McFPS* by using GC–MS (C, γ-muurolene; D, δ-selinene; E, *cis*-β-farnesene; F, δ-neoclovene) Note: M: DNA marker; lines 1–2: non-transgenic control callus tissues; lines 3–6: independent putative transformants of resistant callus tissues. Error bars are shown with three biological replicates; ** indicates a very significant difference (*p* < 0.01). The 18S gene was used as the reference gene. The 2^−ΔCt^ method was used to calculate relative expression levels

Because the hygromycin gene serves as a resistance gene in the vector pCAMBIA1302, resistant callus tissues were first identified using PCR with hygromycin gene-related primers. The results indicated an approximately 750 bp fragment, which is the same size as the hygromycin gene fragment in resistant callus tissues, and this fragment was absent in the non-resistant callus tissue (Fig. 4A). Moreover, the expression level of *McFPS* was 5 times higher in the resistant callus tissues than in the non-resistant callus tissues, which indicated that *McFPS* had successfully transferred to the callus tissues of German chamomile (Fig. 4B). Furthermore, sesquiterpenes were detected in the transgenic callus tissues by using GC–MS. The results showed that sesquiterpenes were more abundant in the resistant callus tissues with transgenic *McFPS* than in the callus tissues with vector pCAMBIA1302. For example, the abundance of γ-muurolene was 60 times higher in the resistant callus tissues with transgenic *McFPS* than in the control group (Fig. 4C). The abundance of δ-selinene was 7.1 times higher in the resistant callus tissues with transgenic *McFPS* than in the control group (Fig. 4D), *cis*-β-farnesene was 3.1 times higher in the resistant callus tissues with transgenic *McFPS* than in the control group (Fig. 4E), and δ-neoclovene was 11.4 times higher in the resistant callus tissues with transgenic *McFPS* than in the control group (Fig. 4F).

### Overexpression analysis of *McFPS* in hairy roots

**Fig. 5:**
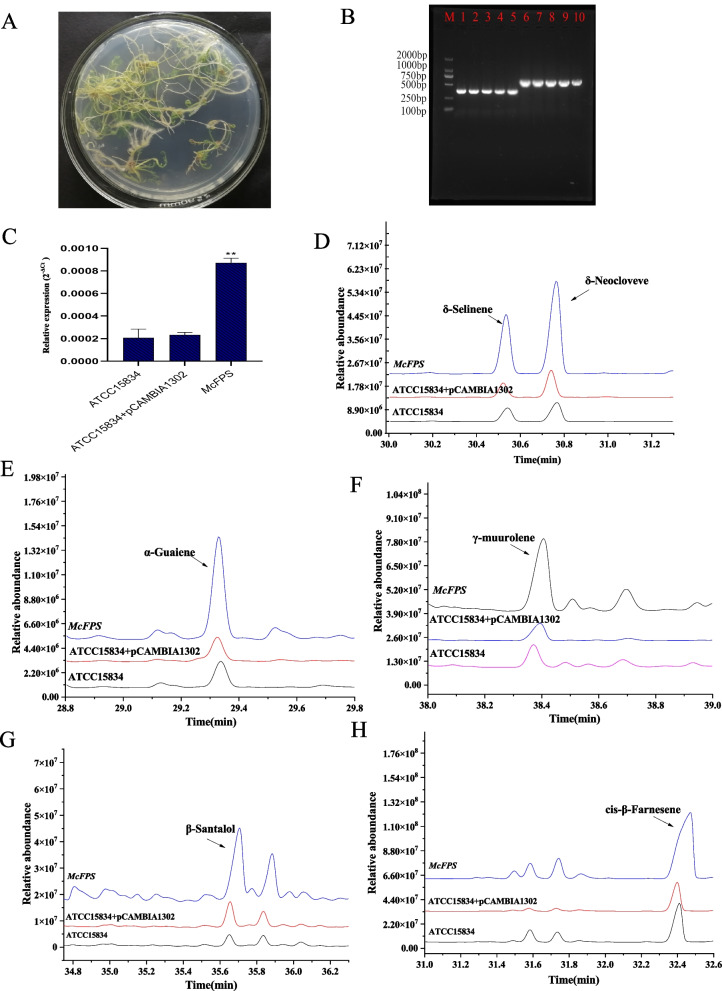
Contents of terpenoids in hairy roots of McFPS detected using GC–MS **A** Transgenic hairy roots of German chamomile. **B** Electrophoresis of rolB and rolC from transgenic hairy roots. **C** qPCR analysis of *McFPS* transgenic hairy roots. **D**–**H** Analysis of volatile components in *McFPS* transgenic hairy roots by using GC–MS (D, δ-selinene and β-neoclovene; **E**, α-guaiene; F, γ-muurolene; **G**, β-santalol; H, *cis*-β-farnesene) Note: M: DNA marker; 1–5: rolB electrophoresis of transgenic hairy roots; 6–10: rolC electrophoresis of transgenic hairy roots. The 18S gene was used as the reference gene. The 2^−ΔCt^ method was used to calculate relative expression levels

To validate the effectiveness of the callus transformation system of German chamomile, we transferred *McFPS* into the hairy roots of German chamomile (Fig. 5A). *rolB* and *rolC* were detected in the transgenic hairy roots, but not in hairy roots transformed with *Agrobacterium rhizogenes* (ATCC 15834) and hairy roots transformed with pCAMBIA1302 empty vector (Fig. 5B). qPCR was used to analyze the gene expression level of *McFPS* in the transgenic hairy roots and controls. The results showed higher transcript levels of *McFPS* in the transgenic hairy roots than in the hairy roots transformed with *A. rhizogenes* (ATCC 15834) and hairy roots transformed with pCAMBIA1302 empty vector (Fig. 5C). GC–MS was used to test for volatiles; notably, overexpression of *McFPS* in the hairy roots led to the accumulation of γ-muurolene, β-santalol, δ-selinene, *cis*-β-farnesene, α-guaiene, etc., which were not detected in the wild-type. The δ-selinene content was 4.2 times higher in *McFPS-*overexpressed hairy roots than in the hairy roots transformed with *A. rhizogenes* and 4.7 times higher in *McFPS-*overexpressed hairy roots than in the hairy roots transformed with pCAMBIA1302 empty vector. The β-neoclovene content was 4.7 times higher in *McFPS-*overexpressed hairy roots than in the hairy roots transformed with *A. rhizogenes* and 3.9 times higher in *McFPS-*overexpressed hairy roots than in the hairy roots transformed with pCAMBIA1302 empty vector (Fig. 5D). The α-guaiene content was 5.8 times higher in *McFPS-*overexpressed hairy roots than in the hairy roots transformed with *A. rhizogenes* and 6.1 times higher in *McFPS-*overexpressed hairy roots than in the hairy roots transformed with pCAMBIA1302 empty vector (Fig. 5E). The γ-muurolene content was 3.8 times higher in *McFPS-*overexpressed hairy roots than in the hairy roots transformed with *A. rhizogenes* and 4.5 times higher in *McFPS-*overexpressed hairy roots than in the hairy roots transformed with pCAMBIA1302 empty vector (Fig. 5F). The β-santalol content was 9.2 times higher in *McFPS-*overexpressed hairy roots than in the hairy roots transformed with *A. rhizogenes* and 4.1 times higher in *McFPS-*overexpressed hairy roots than in the hairy roots transformed with pCAMBIA1302 empty vector (Fig. 5G). The *cis*-β-farnesene content was 2.8 times higher in *McFPS-*overexpressed hairy roots than in the hairy roots transformed with *A. rhizogenes* and 4.1 times higher in *McFPS-*overexpressed hairy roots than in the hairy roots transformed with pCAMBIA1302 empty vector (Fig. 5H).

## Discussion

The *Agrobacterium*-mediated transformation system is an effective method for plant gene functional studies [26], and mechanisms that regulate the expression or transformation levels have been broadly studied in several plant species. For example, Nakajima et al. used the *Agrobacterium*-mediated method to transfer plasmids containing *sgfp* and *nptII* genes into embryonic callus tissues and induce seedlings with a transformation efficiency of nearly 100% [27]. Ebrahimzadegan and Maroufi established an *Agrobacterium*-mediated genetic transformation system for obtaining transgenic plants by using *Lallemantia iberica* cotyledonary nodes as explants [28]. Hayta et al. developed and optimized an *Agrobacterium-*mediated genetic transformation system with efficiencies of up to 25% [29]. German chamomile is an aromatic medicinal plant that belongs to the genus *Matricaria* in the family Asteraceae. The flowers are rich in terpenoids with anti-inflammatory, antioxidant, and bacteriostatic effects, and they are widely used in medicine, cosmetics, and aromatherapy. However, a transformation system has not yet been established for German chamomile.

Many factors, such as co-culture time, infection solution concentration, infection time and antibiotic concentration, affect *Agrobacterium*-mediated genetic transformation. Co-culture time an important factor of genetic transformation. If the co-culture time is short, *A. tumefaciens* cannot complete the transfer of T-DNA. When the co-culture time is too long, overgrowth of *A. tumefaciens* may occur, which greatly reduces plant viability and transformation efficiency. Utami et al. found that the highest infestation efficiency was achieved when the explants were co-cultured with the strain for 5 days [30]; the lowest infestation efficiency was achieved when the explants were co-cultured with the strain for 1 day. In this study, the optimum co-culture time of German chamomile was 3 days, and the transformation effect was the best and transformation rate was the highest.

**Fig. 6:**
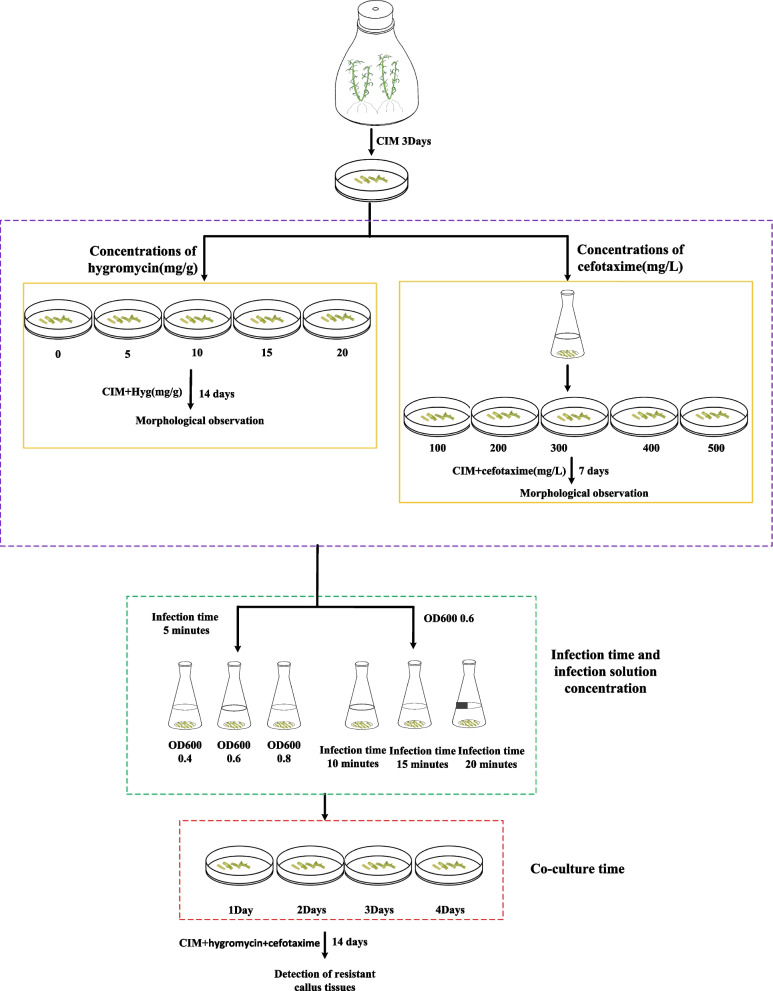
The schematic diagram of the establishment callus tissue transformation system for German chamomile

*A. tumefaciens* infestation time and infection solution concentration are important factors that affect the transformation efficiency. If the infiltration time is too short or the concentration of bacterial solution is too low, *A. tumefaciens* may show insufficient adhesion and the recombinant plasmid cannot be efficiently transferred into plant cells, which greatly reduces the transformation efficiency. Similarly, if the bacterial solution concentration is high or the infiltration time is long, *A. tumefaciens* adheres to the explants in large numbers, which reduces the transformation efficiency. Ma et al. suggested that the cell density of *A. tumefaciens* was the highest at OD_660_ = 0.8 and 30 min of infiltration for callus tissue transformation [31]. In this study, we found that the suitable bacterial concentration and infection time for German chamomile were OD660 = 0.6 and 5 min, respectively. The selection of antibiotic concentration is the key step to obtain transgenic plants. Prabin et al. demonstrated that *Chlorella* can be screened at a hygromycin concentration of 30 mg/L [32]. Kim et al. found that the effect of transgenic screening was the best when K^+^ concentration was 125 mg/L [33]. In this study, we found that the efficiency of resistant callus tissues was the highest when hygromycin concentration was 10 mg/L. Furthermore, when cefotaxime concentration was 300 mg/L, the growth of *A. tumefaciens* was completely inhibited and callus tissue growth was normal. In this research, a successful genetic transformation system for German chamomile callus tissue was established, providing a foundational platform for validating gene functions in German chamomile [34, 35] (Fig. 6).

Hairy roots are highly differentiated and have the entire system of secondary metabolite synthesis of parental plants, and they can be genetically engineered to produce a large number of secondary metabolites and substances not originally contained in the roots. Recently, some important plant secondary metabolites (ginsenosides, saponins, alkaloids, and artemisinins) have been studied in vitro in trichome roots [36]. Ding et al. overexpressed transcription factor *SmMYB36* in trichome roots to promote the accumulation of tanshinone in large quantities [37]. Hidalgo et al. used wild tobacco trichome roots to produce the anti-cardiovascular disease drug resveratrol [38]. Renouard et al. investigated the production of anticancer lignans from a cultured strain of the trichome root of *Linum usitatissimum* [39]. In a previous study, a German chamomile trichome root transformation system was established, and it was used for terpenoid pathway studies. In this study, we obtained transgenic callus tissues and trichome roots for *McFPS* by using the genetic transformation system and trichome induction system of German chamomile. The GC–MS assays of the transgenic callus tissues and hairy roots showed a significant increase in primary sesquiterpene material when compared with the control. The hairy root genetic transformation system and callus tissue genetic transformation system can together verify gene function. Advantages of the callus tissue genetic transformation system of German chamomile are its easy operation, high stability, reduced complexity of transgenic plant screening, and shorter induction time, for example, transgenic callus tissues can provide results in a short time (about 20 days), whereas hairy roots take longer. Thus, in this study, a stable, universal, and widely usable callus tissue genetic transformation system was developed for transgenic German chamomile plants. The results lay the foundation for further gene editing and gene function studies of German chamomile.

## Conclusion

The genetic transformation system is important for plant quality improvement and molecular research. However, to the best of our knowledge, an efficient genetic transformation protocol for German chamomile has not yet been reported. In this study, we used the petiole of German chamomile as explant material; optimized the bacterial solution concentration, hygromycin concentration, cefotaxime concentration, co-culture time, and infection duration; and confirmed that the system could be stably. The German chamomile callus tissue genetic transformation system established in this study will lay the foundation for future gene function validation of German chamomile.

## Materials and methods

### Plant material

German chamomile seedlings were obtained from Anhui Cishou Botanical Factory Co., Ltd. and planted at the experimental farm of Anhui Agricultural University (Hefei City, Anhui Province, China). They were sown at the beginning of March and grew to about June under natural conditions. Samples from different flowering stages and organs were randomly selected and immediately frozen in liquid nitrogen and stored at 80℃ for subsequent RNA and DNA extraction. Three biological replicates were used per sample. After German chamomile bloomed, the mature seeds were collected and stored for tissue culture experiments.

### RNA and cDNA synthesis

Total RNA was extracted from the disk florets of German chamomile during the full-blossom period by using an RNA extraction kit (Tiangen, Beijing). The concentration, purity, and integrity of total RNA were determined using the nucleic acid protein quantitation instrument and agarose gel electrophoresis [40]. The RNA was reverse-transcribed using a reverse transcription kit (TransGen Biotech, Beijing, China), and the cDNA was stored at -20℃.

### Cloning the cDNA of *McFPS*

On the basis of our laboratory transcriptome data for German chamomile, *McFPS* was selected as the candidate gene. The NCBI website (https://www.ncbi.nlm.nih.gov/) was used to conduct sequence alignment [41]. Specific primers were designed using Primer Premier 5 software (Table S1), and the 5′-terminal and 3′-terminal sequences of *McFPS* were obtained using the SMARTerR RACE 5′/3′ Kit User Manual Kit (TaKaRa). The amplified fragments were compared with sequences in the NCBI database to determine the accuracy of the amplified fragments. The full-length sequences of *McFPS* were obtained by sequence splicing with DNAMAN 8.0 software. Specific primers were designed to amplify the full-length cDNA of *McFPS*. The amplicons were connected to the T_3_ vector and sequenced.

### Selection of explants

The seeds of German chamomile were soaked in carbendazim solution for 12 h, cleaned with water, dried in a drying oven at 37℃, and sterilized in a closed drying dish with 90 mL NaClO solution and 3 mL HCl gas for 12–16 h. The seeds were germinated on MS medium at 25 ± 2℃ in the light. Seedlings that grew for about 2 months were selected [42]. We selected petiole segments of German chamomile as explants and cultured the explants on CIM for 3 days.

### Hygromycin lethality test

To establish an appropriate hygromycin concentration for selecting transformed callus tissues, petiole segments of German chamomile were cultivated on CIM with different concentrations of hygromycin (0, 5, 10, 15, and 20 mg/L). The petiole segments were cultivated in petri dishes, with three replicates for each hygromycin concentration. The browning rate was evaluated after 14 days of cultivation.

### Effect of cefotaxime on *A. tumefaciens*

The petiole segments of German chamomile were cultured on CIM with different concentrations of cefotaxime (100, 200, 300, 400, and 500 mg/L) after *A. tumefaciens* infection. The petioles were cultured in petri dishes, with three replicates for each cefotaxime concentration. The contamination rate was measured after 7 days of culture.

### Effects of infection time and infection concentration on callus tissue induction

On the basis of the optimized concentrations of hygromycin and cefotaxime, *McFPS* was constructed into the plant expression vector pCAMBIA1302 and transferred into *Agrobacterium tumefaciens* EHA105, which was then used to transform the German chamomile explants. The EHA105-containing recombinant plasmid was transferred to LB plates with 50 μg/mL kanamycin and 20 μg/mL rifampicin for 2 days at 28℃; then, a single colony was transferred to 100 mL LB for 1–2 days at 28℃, and centrifuged for collection. The collected thalli were suspended in 1/2 MS liquid medium containing 100 μmol/L acetosyringone (As). OD_600_ of the bacterial suspension was measured using a spectrophotometer with 1/2 MS medium as the blank. The petioles were then infected by soaking them in *A. tumefaciens* suspensions of different concentrations (OD_600_ = 0.4, 0.6, and 0.8). The petioles were infected for varying periods (5, 10, 15, and 20 min) at room temperature. The resistant callus tissue rate was analyzed and counted after 30 days of culture.

### Effects of co-culture time on the transgenic callus tissue culture of German chamomile

The influence of co-culture duration on the transgenic callus tissue culture of German chamomile was observed under the conditions mentioned previously. The explants were washed with sterile distilled water to remove excess bacteria, blotted dry on a sterile filter paper, and subsequently transferred to CIM. Then, the petioles of German chamomile were cultured on resistant callus induction (MS + 2.0 mg/L 6-BA + 0.4 mg/L NAA + 300 mg/L cefotaxime + 10 mg/L hygromycin) for 1, 2, 3, and 4 days at 25 ± 2 °C in the dark. The transformation efficiency of resistant callus tissues was recorded.

### Identification of transgenic callus tissues

Genomic DNA was isolated from callus tissues transformed with pCAMBIA1302 empty vector and callus tissues transformed with *McFPS* by using a plant DNA extraction kit (Biomiga, China), according to the manufacturer’s instructions. Hygromycin resistance genes were amplified with PCR by using specific primers and Phanta Max Super-Fidelity DNA Polymerase (Vazyme, Nanjing). The PCR conditions were as follows: initial denaturation at 95℃ for 3 min, followed by 35 cycles at 95℃ for 15 s, 60℃ for 15 s, and 72℃ for 30 s and a final extension step at 72℃ for 5 min. For gene expression analysis, total RNA was extracted from the calli by using an RNA plant extraction kit (Tiangen, Beijing) and reverse-transcribed using a reverse transcription kit (TransGen Biotech, Beijing, China), according to the manufacturer’s instructions. qPCR amplification of the cDNA was performed using SYBR Green qPCR Mastermix (Vazyme, Nanjing) and qPCR primers [43]. The 18S rRNA gene was used as the reference gene. All qPCR analyses were performed with three biological and three technical replicates. Callus tissues transformed with pCAMBIA1302 empty vector were used as controls.

### Overexpression of *McFPS* in German chamomile hairy roots

To confirm the effectiveness of the German chamomile callus transformation system, we constructed recombinant plasmids pCAMBIA1302-McFPS and transfected the recombinant plasmids into rootless German chamomile seedlings with *A. rhizogenes* to induce hairy roots [42]. The method was as follows: First, German chamomile seeds were cultured on MS medium at 25 ± 2 °C in the light after sterilization. Second, roots of one-month-old German chamomile plants were pruned away, and the wounds were infected with *A. rhizogenes* (ATCC 15834) containing the recombinant plasmids pCAMBIA1302-McFPS. Third, the explants were cultivated on MS medium for co-cultivation with *A. rhizogenes* and then on B5 medium containing cefotaxime (500 mg/L) and hygromycin (20 μg/mL). Lastly, transgenic hairy roots were identified and analyzed after 6 weeks. *A. rhizogenes* contains the Ri plasmid; *rolB* and *rolC* are specific gene sequences in the T-DNA region of the Ri plasmid, which can maintain hairy root morphology and growth. The presence of *rolB* and *rolC* was detected using PCR. The expression level of *McFPS* in the hairy roots was analyzed using qPCR. The 2^−ΔCt^ method was used to calculate the relative expression levels of *McFPS*, and the 18S rRNA gene was used as the reference gene. German chamomile hairy roots transformed with pCAMBIA1302 empty vector were used as controls. Lastly, 0.3 g of transgenic hairy roots and 0.3 g of hairy root transformed with pCAMBIA1302 empty vector were collected for GC–MS. Hairy roots transformed with *A. rhizogenes* (ATCC 15834) and hairy roots transformed with pCAMBIA1302 empty vector were used as controls.

### GC–MS analysis

Changes in the volatile components of transgenic callus tissues and hairy roots were detected using GC–MS with an Agilent 7000B instrument (Agilent, California, USA). Samples (0.3 g) were cut into pieces and placed in headspace bottles and incubated in a water bath for 50 min at 80 °C. After allowing the sample and headspace to equilibrate, the SPME fiber was inserted into the headspace for 30 min at room temperature (30 °C) for extraction of volatiles [44]. After extraction, the SPME fiber was inserted into the GC port (Agilent 7890B-7000B) and subjected to thermal desorption at 250℃ for 10 min; then, the compounds were separated using an HP-5MS column (30 m × 0.25 mm I.D. × 0.25 μm film thickness; Agilent Technologies, USA) [45]. The carrier gas was high-purity helium (99.999%, Airgas Inc.); the flow rate was 1 mL/min. The injector temperature was 250 °C, and the injection mode was splitless. The oven temperature was maintained at 40 °C for 5 min, increased to 250 °C at a rate of 10 °C per minute, and subsequently held at 250 °C for an additional 5 min. In MS, the transfer line temperature was set to 280 °C. Electron impact ionization was conducted at 70 eV, the quadrupole was maintained at 150 °C, and the scanning range encompassed 20–400 m/z. The scan rate was set at 3.75 scans per second, and the ion source temperature was 230 °C. Identification of volatile components was conducted by comparing the spectra with those in the National Institute of Standards and Technology database. Hairy roots transformed with *A. rhizogenes* (ATCC 15834) and hairy roots transformed with pCAMBIA1302 empty vector were used as controls. Three replicates of each group were analyzed.

### Data analysis

The data in histograms are means ± standard deviations (SD; *t*-test), and the error bars indicate SDs of three biological replicates. The histograms were prepared using Microsoft Excel 2019 software and GraphPad Prism 8.0.2. Three biological replicates were used per sample. Additional data and materials can be made available upon request. A *P* value < 0.05 was considered statistically significant.

### Supplementary Information

**Additional file 1.**

## Data Availability

All data generated or analyzed during this study are included in this published article and its supplementary materials.
